# Analyzing the Status of Sustainable Development in the Manufacturing Sector Using Multi-Expert Multi-Criteria Fuzzy Decision-Making and Integrated Triple Bottom Lines

**DOI:** 10.3390/ijerph17113800

**Published:** 2020-05-27

**Authors:** Sepehr Hendiani, Huchang Liao, Morteza Bagherpour, Manuela Tvaronavičienė, Audrius Banaitis, Jurgita Antucheviciene

**Affiliations:** 1 Department of Industrial Engineering, Iran University of Science and Technology, Tehran 50727322, Iran; Sepehr.hendiani@gmail.com (S.H.); Bagherpour@iust.ac.ir (M.B.); 2 Business School, Sichuan University, Chengdu 610064, China; 3 Department of Business Technologies and Entrepreneurship, Vilnius Gediminas Technical University, 10223 Vilnius, Lithuania; manuela.tvaronaviciene@vgtu.lt; 4 General Jonas Zemaitis Military Academy of Lithuania, 10223 Vilnius, Lithuania; 5 Department of Construction Management and Real Estate, Vilnius Gediminas Technical University, 10223 Vilnius, Lithuania; audrius.banaitis@vgtu.lt (A.B.); jurgita.antucheviciene@vgtu.lt (J.A.)

**Keywords:** sustainability and lifestyles, manufacturing companies, MCDM, environmental sustainability, fuzzy decision making

## Abstract

A sustainable manufacturing company depends on the developments in three aspects in order to minimize harmful impacts on the environment, improve the social relations, and simultaneously maximize the economic benefits. Despite the increasing types of investigations that researchers have carried out in environmental and economic aspects, the minimum attention has been paid to social relations. In response to this deficiency, this paper proposes a new framework to obtain the overall sustainability index in manufacturing companies by encapsulating the sustainability criteria/sub-criteria. This article collected 33 sub-criteria for five pillars of sustainability as social, environment, economic, technological advancement, and performance management. The key contributions of this paper are highlighted as the hierarchical method that obtains the status of sustainability in uncertain conditions, the ability to identify the weak points, and a new framework for gathering the data about sustainability performance in manufacturing companies. The findings of this paper will aid both policymakers and decision-makers to assess the sustainability status of manufacturing systems and improve the performances of them.

## 1. Introduction

Sustainable development implies providing the way of meeting the needs by utilizing the maximum ability of current generation without compromising the abilities and technologies of future generation [1,2]. Sustainable enhancement depends on augmenting three aspects including social, economic, and environment which are called sustainability pillars [3,4]. As a practical contribution, sustainability aims at minimizing the harmful impact on the environment, promoting good social relations and increasing the benefit at the same time.

In the past decade, the increasing pressure of government regulations concerning manufacturing companies forced them to consider sustainability insights and think beyond only economic benefits [5,6,7]. These companies have been motivated to consider other sustainability aspects including social and environmental perspectives. It has thus turned into a goal for manufacturing companies to promote such practices that minimize the environmental impacts and simultaneously enhance the social and economic benefits. Hence, this desire motivated manufacturers and manufacturing companies to increase their chance to gain a share in the competitive market place by regulating some rules towards implementing sustainability practices in developed and developing countries [8]. Given this, researchers and scholars tried to develop methods [9,10,11,12] and scientific approaches which aim at enhancing the sustainability level in manufacturing companies by recognizing the potential of the associated system and proposing corresponding improvements [13,14].

Sustainable manufacturing strategy involves three stages: (I) selection of appropriate criteria for measuring sustainability of manufacturing company, (II) a suitable assessment tool which determine the sustainability status and identify the fragile points, and (III) modifications of different parts of the system to enhance the sustainable manufacturing subsequently [15,16]. Regarding the abundant studies that carried out with the purpose of enhancing the total sustainability in manufacturing companies, less attention was always paid to determining the current status of sustainability in the system. Most of the previous studies utilized appropriate attributes to identify weak points and improving the quality of them [16,17,18,19]. In order to determine the condition of attributes, decision-makers need to state a generic term, e.g., very high, which is derived from a subjective preference and involves uncertainty indeed. Regarding this, the fuzzy theory has been utilized by researchers to quantify these subjective expressions. Since the sustainability researchers investigated the usefulness of fuzzy theory, they have incorporated this concept with evaluations of sustainability [20,21,22,23,24].

This study proposes a new benchmark approach which aims at measuring the current level of sustainability in manufacturing systems, identifying the weak points that have a negative impact on overall sustainability level and enhancing the efficiency of weak points to uplift the overall sustainability index subsequently. The fuzzy logic is utilized to consider the uncertainty and help decision-makers to express their subjective preferences by linguistic variables. Note that the fuzzy membership function is also utilized to transfer these linguistic terms. The application of manufacturing companies’ sustainability triple bottom line (TBL) criteria/sub-criteria, trapezoidal fuzzy set’s principles and fuzzy aggregation operator helped us in introducing a new as fuzzy sustainable manufacturing company index (FSMCI).

We highlight the contributions and innovations of this study as follows:

➢ The most appropriate manufacturing companies’ sustainability TBL criteria are collected from the extended literature to expose a holistic framework which adds to the state-of-the-art studies of sustainability in manufacturing sector by encapsulating a proposal and applications of a new and fuzzy sustainable manufacturing company index.
➢ The performance indicator abbreviated as FPII is utilized to identify the weak performances concerning sustainability TBL criteria which can help DMs immensely to improve the quality of weak points or so-called obstacles and consequently enhance the overall sustainability level.
➢ The trapezoidal fuzzy membership functions are employed which reflect more accurate results [25]. A new trapezoidal fuzzy ranking model is also utilized to compare the performance of every single sub/criteria with a managerial threshold. Note that fuzzy aggregation operator is used to obtain the sustainability of each level including sub-criteria level, criteria level, and finally the overall sustainability index (after criteria level).
➢ The following approach proposes a new method by which decision makers can decide about the performance of every sub-criteria based on the opinion of more than one expert. In other words, the mean of judgments will be set as the performance of sub-criteria which is obviously more reasonable than the opinion of one DM. However, the importance weights of criteria/sub-criteria will be determined based on the objectives of the organization.

The remainder of this study is organized as follows: in Section 2, the state-of-the-art literature of sustainable manufacturing companies and adopted approaches are reviewed. Section 3 provides the modeling procedure by expressing the step-by-step principles. In Section 4, an illustrative case is proposed with step-by-step illustrations to explain the wide application of the proposed approach in real-world manufacturing sectors. Section 5 and Section 6 indicate the discussions and conclusions, respectively.

## 2. Literature Review

In this section, the up-to-date literature of sustainability in manufacturing companies and the application of fuzzy sets in sustainability context are reviewed which helps with illustrating the necessity of implementation and wide applications of the proposed approach in real-world manufacturing companies. Note that authors tried to sieve these reviewed studies despite the fact there are numerous masterpiece works in the context of sustainable manufacturing.

### 2.1. Measuring Sustainability Using Fuzzy Logic

This sub-section provides a comprehensive scheme of the methods and approaches which are developed for evaluating sustainability in a diverse industry by the usage of fuzzy theory. The fuzzy theory defined by Zadeh [26] opened a door to uncharted territory. These types of sets were introduced to transfer linguistic terms into a set of number which assists decision-makers to incorporate uncertainty. As a linguistic-based approach, the fuzzy sets are suitable for cases where the sensitivity of inputs plays a significant role on the accuracy of outputs and the final decision. With the increasing attention to the implementation of fuzzy sets, many scholars have utilized fuzzy theory to model uncertainty in decision-making problems [21,27,28,29,30,31,32,33,34]. Some of these studies have been reviewed below.

Lin et al. [35] proposed a model for obtaining a total index by aggregating a fuzzy operator on agility sub-criteria, criteria, and enablers. Given that, a three-level calculation method determined the fuzzy agility index for a specific corporation. Rajak and Vinodh [11] developed that approach by collecting social sustainability attributes, criteria, and enablers and applying a similar approach to obtain a fuzzy social sustainability index for an automotive component manufacturing company. Furthermore, Rajak et al. [36] generalized their social sustainability measurement approach by moving towards total sustainability analysis, which integrated three sustainability TBL attributes at the same time. They adopted the approach proposed by Lin et al. [35] to obtain a fuzzy sustainability index for the transportation industry. Kumar and Anbanandam [21] also adopted the same approach to determine the social sustainability level of the freight transportation industry by proposing a conceptual framework which consists of social sustainability enablers, dimensions, and attributes concerning freight transportation organizations. Singh et al. [37] proposed a methodology for evaluating sustainability in small and medium-sized enterprises (SMEs) using fuzzy AHP, fuzzy inference systems, and balanced scorecard. They collected performance indicators from the literature review and proposed a generic framework in which decision-makers determined the performance ratings and importance weights based on linguistic inputs. Ocampo et al. [38] proposed a model which explores the significance of stakeholder’s interests when developing a manufacturing strategy using fuzzy logic. Note that all the studies mentioned have utilized triangular fuzzy membership functions to transfer linguistic terms and yet the trapezoidal fuzzy sets have remained intact.

Phillis et al. [39] modified the sustainability evaluation by a fuzzy model, which was proposed to assess the sustainable development of countries, to appraise the sustainable development of all the cities in the world. They defined sustainability as a function of two pillars including ecological and well-being. The third pillar or economic is placed in well-being with regard to other sub-criteria including education, health, and civic environment of cities. The ecological input depends on the state of air, water, land, and other environmental attributes. Kouikoglou and Phillis [40] proposed a fuzzy-based corporate sustainability analysis by combining normalized inputs, which are prioritized based on their sustainability status, to obtain an overall sustainability index on [0,1]. The most important criteria and sub-criteria that utilized in the mentioned studies are collected to establish the framework of this study.

Another topic which is widely utilizing the application of fuzzy theory is the sustainable supplier selection problem. These past years, a huge number of studies have been carried out with the background of fuzzy sustainable supplier selection which mostly utilized fuzzy decision-making approaches [41,42,43,44]. A sustainable supplier selection refers to selecting the best supplier which adhere to regulations about social, economic, and environmental dimensions at the same time. Yu et al. [41] proposed a framework in which sustainability TBL attributes are collected and weighted by using Pythagorean fuzzy sets which are a generalization for conventional fuzzy sets. Memari et al. [45] developed a multi-criteria intuitionistic fuzzy TOPSIS method which determined the weight of sustainability TBL attributes and ranked the suppliers based on the performance of corresponding attributes. Awasthi et al. [46] proposed five dimensions for sustainable suppliers including economic, social, environmental, quality, and global risk. They also developed a fuzzy AHP-VIKOR approach for evaluating sustainable suppliers involving sustainability risks from sub-suppliers.

### 2.2. Sustainability in Manufacturing Companies

Due to the fact that manufacturing companies are playing an active role in the consumption of natural resources and generation of waste materials, they can significantly contribute to promoting sustainability development in developed/developing countries [47,48]. Regarding the increasing amount of environmental impacts caused by manufacturing companies, most of the studies have investigated the environmental aspect of sustainability through manufacturing companies and factories [49,50,51].

Mostafa and Dumrak [52] proposed a framework for controlling waste in manufacturing companies with three consecutive phases including waste documentation, waste analysis, and waste removal. The study identified nine types of waste in manufacturing processes. Muñoz-Villamizar et al. [53] and Vegera et al. [54] presented how manufacturing companies carry out manufacturing and environmental practices. They also proposed some reflections for correctly measuring the environmental efficiency in manufacturing companies. Shashi et al. [55] explored the hypothesized model empirically utilizing data from 374 Indian manufacturing companies to investigate the relationships between leanness, financial performance, product innovation, process innovation, and environmental performance.

Meanwhile, there are also a few studies which integrated sustainability pillars to propose assessment models for manufacturing companies by encapsulating related criteria, sub-criteria, and attributes. A selection of these studies is reviewed in Table 1 to help in collecting the sub-criteria for the proposed framework. Note that the following indicators are collected as a generic framework and can be changed based on the objectives and nature of manufacturing companies. The following framework consists of five categories including Social, Economic, Environmental, Technological advancement, and Performance management [8].

## 3. The Methodology for the Sustainability Assessment of Manufacturing Companies

In this section, first, the basic concepts that are utilized for elaborating this study are briefly denoted based on original references. After that, these concepts are merged to establish the proposed methodology.

**Figure 1:**
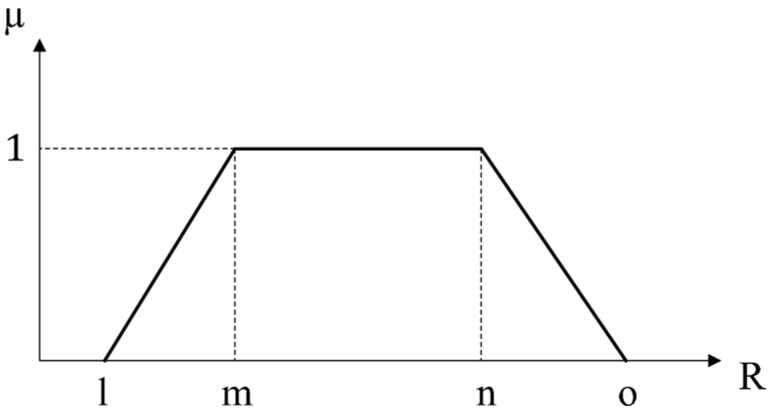
The trapezoidal fuzzy membership function.

Regarding the type of fuzzy set which is incorporated for modeling the sustainability assessment in this study, some basic definitions are explained to provide a comprehensive illustration of trapezoidal fuzzy sets (TrFS). The TrFS are utilized instead of triangular fuzzy sets to cover more uncertainty and conclude more accurate results [25,59,60]. A conceptual definition of trapezoidal fuzzy sets is denoted as follows:

Assume that $\tilde{A}=\left[l,m,n,o\right]$ is a trapezoidal fuzzy number. The trapezoidal membership function is defined as Equation (1). Figure 1 demonstrates the membership function of A [61].

(1) $$\mu_{\tilde{A}}(x)=\left\{ \begin{array}{ll} 0 & x<l\\ \frac{x-l}{m-l} & l<x<m\\ 1 & m<x<n\\ \frac{x-n}{o-n} & n<x<o\\ 0 & x>o \end{array}\right.$$

It should be noted that a trapezoidal fuzzy membership can be converted to a simple triangular fuzzy membership if m=n.

Assume that $\tilde{A}=\left[a_{1},a_{2},a_{3},a_{4}\right]$ and $\tilde{B}=\left[b_{1},b_{2},b_{3},b_{4}\right]$ are two trapezoidal fuzzy numbers. The basic operations of these two fuzzy set are defined as follows [26]:

(2) $$\tilde{A}+\tilde{B}=\left[a_{1}+b_{1},a_{2}+b_{2},a_{3}+b_{3},a_{4}+b_{4}\right]$$

(3) $$\tilde{A}-\tilde{B}=\left[a_{1}-b_{4},a_{2}-b_{3},a_{3}-b_{2},a_{4}-b_{1}\right]$$

(4) $$\tilde{A}\times \tilde{B}=\left[a_{1}b_{1},a_{2}b_{2},a_{3}b_{3},a_{4}b_{4}\right]$$

(5) $$\tilde{A}/\tilde{B}=\left[a_{1}/b_{4},a_{2}/b_{3},a_{3}/b_{2},a_{4}/b_{1}\right]$$

A fuzzy number $\tilde{A}$ in parametric form is a pair $\left(\underline{A},\bar{A}\right)$ of functions $\underline{A}\left(r\right)$, $\bar{A}\left(r\right)$, 0<r<1 which satisfy the following requirements [62]:

1. $\underline{A}\left(r\right)$ is a bounded monotonic increasing left the continuous function
2. $\bar{A}\left(r\right)$ is a bounded monotonic decreasing right continuous function
3. $\underline{A}\left(r\right)\leq \bar{A}\left(r\right)$, 0<r<1

For an arbitrary trapezoidal fuzzy number $\tilde{A}=\left[a_{1},a_{2},a_{3},a_{4}\right],=\left(x_{0},y_{0},\sigma ,\beta \right)$ with parametric form $\left(\underline{A},\bar{A}\right),$ the magnitude of $\tilde{A}$ will be defined as follows [62]:

(6) $$Mag\left(\tilde{A}\right)=\frac{1}{2}\left(\int_{0}^{1}\left(\underline{A}\left(r\right)+\bar{A}\left(r\right)+x_{0}+y_{0}\right)f\left(r\right)dr\right)$$

While $x_{0}=a_{2}$, $y_{0}=a_{3},\;\sigma =a_{2}-a_{1},\;\mathrm{and}\;\beta =a_{4}-a_{3}$, we have $\underline{A}\left(r\right)=x_{0}-\;\sigma +\;\sigma r$ and $\bar{A}\left(r\right)=y_{0}+\;\beta -\;\beta r$.

The main purpose of this paper is to improve the efficiency of sustainability practices in manufacturing companies by determining the current status of sustainability, identifying obstacles and improving them. This goal will be achieved by improving the quality of every single sub-criteria indeed. The perfection of these criteria directly depends on the selection of appropriate sub-criteria.

**Figure 2:**
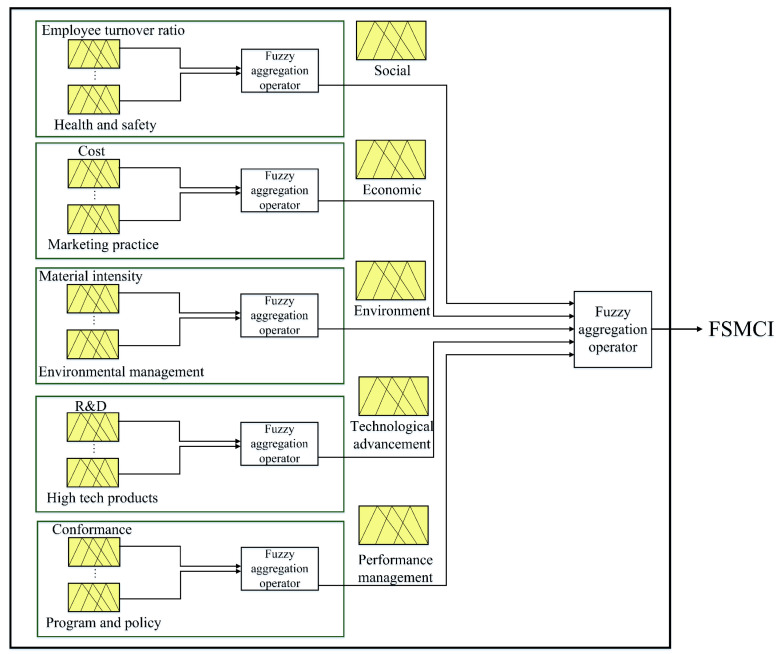
The proposed sustainability assessment model.

Table 1 indicates the most common sub-criteria collected from the literature review to address the leading aspects that are needed to be assessed in a sustainable manufacturing company. The proposed sustainability assessment framework is shown in Figure 2.

This paper presents a trapezoidal fuzzy sustainability assessment model to obtain a trapezoidal fuzzy sustainable manufacturing company index. Given that, a convertor table is required which transfers the linguistic terms by corresponding trapezoidal fuzzy sets. Table 2 can be a sound basis for transferring these linguistic terms for both performance rates and importance weights.

The steps of implementing the proposed approach have been explained with details as follows:

Step 1: Select the most suitable criteria and sub-criteria regarding the objectives of the company and establish an appropriate questionnaire with linguistic answers shown in Table 2. In addition, determine the weight of selected criteria/sub-criteria using the linguistic preference of stakeholders, shareholders, etc. use Table 3 to select the suitable sub-criteria.

Step 2: Use the judgments of experts/decision-makers to determine the performance rate of each sub-criterion using the linguistic terms shown in Table 2 again. Note that, for more than one decision-maker, the mean of judgments has to be obtained once the linguistic terms are transferred to trapezoidal fuzzy sets.

Step 3: Obtain the fuzzy sustainable manufacturing company index using the fuzzy aggregation operator [21] as Equation (7). Note that this aggregation operator obtains the performance rate of the next level. By starting in the sub-criteria level and importing importance weights and performance rates to Equation (7), the performance rates of the next level (criteria level) will be obtained. Using the same equation for the criteria level will obtain the final FSMCI index:

(7) $$R_{i}=\frac{\sum^{\;}W_{\mathrm{ij}}\times R_{\mathrm{ij}}}{\sum^{\;}W_{\mathrm{ij}}}\;\mathrm{where}\sum^{\;}W_{\mathrm{ij}}=1,$$

where $R_{\mathrm{ij}}$ and $W_{\mathrm{ij}}$ represent the performance rating and importance weight of the jth sub-criteria of the ith criteria, respectively.

Once the FSMCI obtained, use Equation (8) to obtain the Euclidean distances [35] between FSMCI and Sustainability Status Terms (SST) to determine the current status of sustainability in the company. The corresponding status of minimum distance will be announced as the status of sustainability:

(8) $$D\left(FSMCI,SST_{i}\right)={\left\{\underset{x\in u}{\sum }{\left[f_{FSMCI}\left(x\right)-f_{SST}\left(x\right)\right]}^{2}\right\}}^{1/2}$$

where $u=\left\{x_{0},x_{1},\ldots ,x_{m}\right\}$.

Step 4: use the fuzzy performance importance index (FPII) to determine the effect of every single sub-criteria on total sustainability. Identify the barriers through the manufacturing company using FPII and improve them in order to enhance the total sustainability index. For every single sub-criteria, the FPII will be obtained by using Equation (9) as follows [21]:

(9) $${\mathrm{FPII}}_{\mathrm{ij}}=W_{\mathrm{ij}}^{*}⊗P_{\mathrm{ij}}\;W_{\mathrm{ij}}^{*}=\left[1,1,1,1\right]-W_{\mathrm{ij}}$$

where $W_{\mathrm{ij}}$ represents for fuzzy importance weight of the sub-criteria ij. Note that once the FPIIs are obtained, a fuzzy ranking method is required for defuzzifying process. The magnitude of a trapezoidal fuzzy number is defined in Section 3 as Equation (6) which obtains an accurate ranking value for each TrFS.

After obtaining the ranked FPIIs for every single sub-criterion, the ones that are lower than the managerial threshold will be identified as weak points. This managerial threshold also can be determined based on the standards of the company. However, in this study, we considered this threshold as the mean of the obtained FPIIs.

**Figure 3:**
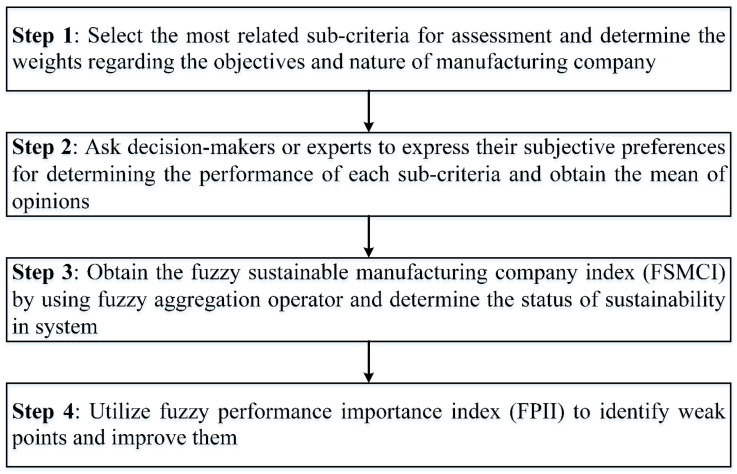
The steps of the proposed methodology.

A guideline illustration of the steps of this method is shown in Figure 3.

## 4. Illustrative Case and Results

In this section, an illustrative case is resolved which totally elucidates the extensive application of the proposed approach. Note that the only difference between this case and the real-world cases is in the collection of data which may differ in diverse cases. Given that, consider a real-world case is being resolved.

The linguistic terms will be offered to DMs and experts, and they will be asked to assign the sub-criteria with a suitable abbreviation, e.g., “UI” for “Utmost importance”.

Hence, consider that the criteria/sub-criteria shown in Table 4 are chosen for this particular manufacturing system and the weights are determined in linguistic form just based on objectives of the company.

Once the sub-criteria and weights are determined, the DMs will cooperate with determining the performance status for each sub-criterion using linguistic terms. For this particular case, four decision-makers are selected to contribute to determine the performances. Table 5 indicates the decisions made by these DMs. The conversion of linguistic terms (refer to Table 2) will indicate the conditions in numerical form.

Table 6 indicates the judgements of every DM and the mean value of these judgements which is calculated for further calculations.

Once the weights and performance rates (mean of DMs’ judgements) obtained, the detailed data will establish Table 7, which shows the weight of both criteria and sub-criteria and also the performance of sub-criteria.

The overall fuzzy sustainable manufacturing company index will be obtained by two levels of calculation using fuzzy aggregation operator Equation (7). This indicator involves the information concerning the status of social, economic, and environmental development. An example of obtaining the performance rating for social criteria is shown below:

$$\begin{array}{l} R_{\mathrm{social}}\\ =\frac{\left[\begin{array}{c} \left[0.5,0.575,0.725,0.8\right]⊗\left[3,3.81,5.43,6.25\right]+\left[0.85,0.9,0.975,1\right]⊗\left[2,2.75,4.25,5\right]\\ +\left[0.5,0.575,0.725,0.8\right]⊗\left[6.5,7.06,8.18,8.75\right]+\left[0.85,0.9,0.975,1]⊗[3.25,4.12,4.56,6.75\right] \end{array}\right]}{\left[\left[0.5,0.575,0.725,0.8\right]+\left[0.85,0.9,0.975,1\right]+\left[0.5,0.575,0.725,0.8\right]+\left[0.85,0.9,0.975,1\right]\right]}\\ =\left[3.41,4.21,5.42,6.59\right] \end{array}$$

Using the same equation, the performance rates for criteria level are obtained and shown in Table 8.

Once the performances of criteria level are obtained, the overall FSMCI index will be calculated using the same equation and based on the results obtained in Table 8:

$$\mathrm{FSMCI}=\frac{\left[\begin{array}{c} \left[0.5,0.575,0.725,0.8\right]⊗\left[3.41,4.21,5.42,6.59\right]+\\ \left[0.5,0.575,0.725,0.8\right]⊗\left[5.77,6.28,7.64,8.23\right]\\ +\left[0.85,0.9,0.975,1]⊗[3.97,4.57,5.75,6.33\right] \end{array}\right]}{\left[\left[0.5,0.575,0.725,0.8\right]+\left[0.5,0.575,0.725,0.8\right]+\left[0.85,0.9,0.975,1\right]\right]}\;=\left[4.30,4.94,6.21,6.99\right]$$

Once the FSMCI is obtained, the Euclidean approach will be utilized to match the FSMCI with sustainability status terms $\left({\mathrm{SST}}_{i}\right)$ to determine the current sustainability status. These status terms are shown in Table 9.

Using the Euclidean distance Equation (8), the distance between FSMCI and the status “extremely sustainable” will be obtained as follows:

$$\begin{array}{ll} D\left(FSMCI,ES\right)= & {\left\{{\left(4.30-7\right)}^{2}+{\left(4.94-7.75\right)}^{2}+{\left(6.21-9.25\right)}^{2}+{\left(6.99-10\right)}^{2}\right\}}^{1/2}\\ & =5.78 \end{array}$$

Using the same equation, the distances will be obtained as shown in Table 10.

## 5. Discussion

Once the distances are all obtained, the minimum distance will be selected as the current status for sustainability in the associated manufacturing company. The results show that the FSMCI has the minimum distance with the status “Normally Sustainable” so this status will be announced for this particular manufacturing company.

To improve the overall sustainability, there is no need for infrastructural modifications, while improving the weak points, they increase the overall sustainability significantly. These improvements firstly depend on the identification of weak points of sustainability.

In this segment, a unified method is elaborated called fuzzy performance importance index (FPII) that help in identifying the obstacles. The FPII of all attributes is calculated and shown in Table 11. As an example, the FPII for sub-criteria “Employee’s training” is computed as follows:

$$W_{ij}^{*}=\left[1,1,1,1\right]-\left[0.5,0.575,0.725,0.8\right]=\left[0.2,0.275,0.425,0.5\right]$$

$${\mathrm{FPII}}_{ij}=\left[0.2,0.275,0.425,0.5]⊗\;[3,3.81,5.43,6.25\right]=\left[0.6,1.04,2.30,3.12\right]$$

To make these trapezoidal fuzzy sets prepared for the sieving process, the ranking score should be obtained for these FPIIs. A ranking method for TrFSs named magnitude of trapezoidal fuzzy set proposed by Abbasbandy and Hajjari [62] which is more accurate than the centroid approach used in previous studies. This approach denoted in Section 3. Using Equation (6), the ranking score for sub-criteria “Employee’s training” will be calculated as follows:

$$x_{0}=1.04,y_{0}=2.30,\sigma =0.44,\beta =0.82$$

$$\underline{u}\left(r\right)=x_{0}-\sigma +\sigma r=1.04-0.44+0.44r=0.6+0.44r$$

$$\bar{u}\left(r\right)=y_{0}+\beta -\beta r=2.30+0.82-0.82r=3.12-0.82r$$

$$Mag\left(u\right)=\frac{1}{2}\left(\int_{0}^{1}\left(\underline{u}\left(r\right)+\bar{u}\left(r\right)+x_{0}+y_{0}\right)f\left(r\right)dr\right)$$

$$\begin{array}{ll} Mag\left(S1\right)= & \frac{1}{2}\left(\int_{0}^{1}\left(0.6+0.44r+3.12-0.82r+1.04+2.30\right)f\left(r\right)dr\right)\\ & \;=\frac{1}{2}\left(\int_{0}^{1}\left(7.06r-0.38r^{2}\right)dr\right)=1.85 \end{array}$$

Using the same equation, the ranking score for every single FPII obtained and shown in Table 11. In this particular case, the managerial threshold is set as the mean of all FPIIs, which is 1.47. Given this, the sub-criteria with ranking scores lower than 1.47 will be identified as weak points in this case. The weak sub-criteria are identified and also shown in Table 11.

**Figure 4:**
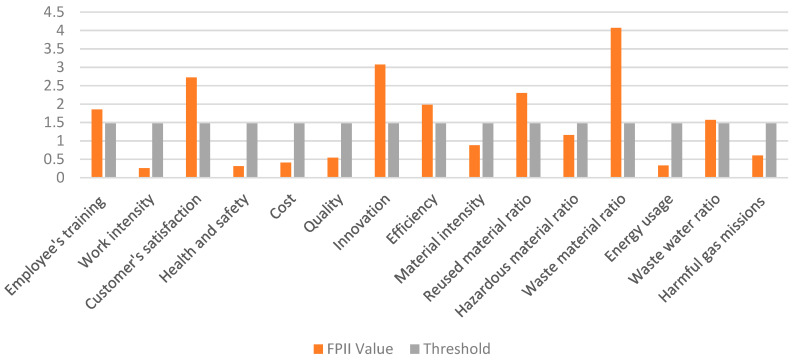
Demonstration of weak points in terms of FPII value.

The weak points can be identified using Figure 4. The values below the threshold and corresponding criteria signify the weak points. The gray bars indicate the threshold value which in this particular case is 1.47. From Figure 4, there are eight weak performing criteria below the threshold. According to the FPII value, “work intensity”, “Cost”, “Health and safety”, and “Energy usage” are the weakest performing criteria, respectively.

Eight sub-criteria out of fifteen seem to be the obstacles of this case. These weak points are distinguished in Table 12. These weak performing criteria can be improved by proper suggestions from managers.

### 5.1. Theoretical and Practical Implications

Following the desire of firms and organizations in performing sustainability practices for remaining in the competitive marketplace, the first step is to determine the current sustainability status in the associated enterprise/system. This study originally adds to the state-of-the-art literature of sustainability in manufacturing systems by encapsulating a proposal and applications of a new and fuzzy sustainable manufacturing company index (FSMCI) which provides a benchmark for sustainability assessments in manufacturing companies.

Meanwhile, this study proposes a conceptual model by accumulating 33 related sub-criteria and classifying them into five criteria of sustainability. Hence, the proposed framework can be considered as a benchmark approach for developing sustainability practices in varied systems. In other words, by collecting related sub-criteria and refreshing Table 1, and also applying the same calculations, the sustainability level of the associated system will be obtained. Note that these sub-criteria can be sieved according to the objectives of the organizations indeed:

The main implications on the theory and practice of this study can be highlighted as follows:

- As a theoretical implication, the proposed model shown in Table 1 can be utilized for collecting related social, economic, and environmental sub-criteria for assessing sustainability in other industries. In addition, the trapezoidal fuzzy membership function and corresponding linguistic terms are shown in Table 2 can be adopted for considering uncertainty in other sustainability assessments.
- Another worthwhile theoretical implication to be mentioned is that it motivates researchers to generalize the proposed method for other industries by following the steps shown in Figure 3. In addition, although it makes the calculations more, weighting methods such as AHP and BWM [63] can be merged with this methodology to make it more in line with real-world data.
- In light of the proposed methodology, the current status of sustainability for manufacturing companies will be determined straightforwardly. Given this, it can be implemented as an entrepreneurship project by involving several scholars and managers as a team for determining/improving the sustainability of manufacturing companies.
- Taking advantage of the proposed framework, policymakers can make policies that enhance the quality of sustainability in manufacturing companies and subsequently there will be quite a lot of advantages obtained including the prevention of waste, reducing the consumption of natural resources, promotion of reusing materials, etc.
- Due to hard computation processes, the previous MCDM approaches were ignored to be utilized in practice. This approach contributes in solving this problem by proposing a soft computing method whilst handling a good amount of uncertainty.

### 5.2. Social and Environmental Implications

According to the vast amount of literature which has been reviewed in the second section, most of the studies considered environmental and economic practices to maximize profit whilst minimizing the harmful impacts on the environment. The findings of this study motivate policymakers to consider social factors such as “work intensity”, “Health and safety”, and “Philanthropy” which have been taken into account less than other factors. In the meantime, this study contributes to consider “Environmental management” as a criterion for environmental aspect of sustainability. This will encourage managers to hire environmental managers and use the knowledge of them to enhance the quality of environmental-oriented practices.

## 6. Conclusions

Despite the increasing amount of research and broad amount of literature with the background of sustainable manufacturing, the development of previous approaches is still a challenging concept. In this study, we proposed an integrated method to assess and obtain the sustainability status in manufacturing companies. In addition, the proposed model conducted a two-layered approach to amend the sustainability index by identifying the weak points in a manufacturing company. This paper developed a generic fuzzy-based assessment approach for measuring the current sustainability status in manufacturing companies by encapsulating thirty-three sub-criteria, collected from the literature and classified in five criteria. The major contributions of the proposed approach can be highlighted as follows:

➢ The model considered five dimensions for sustainability including social, environmental, economic, technological advancement, and performance management. In addition, thirty-three sub-criteria were collected from the literature and have been classified into those five criteria to obtain the fuzzy sustainable manufacturing company index (FSMCI).
➢ The trapezoidal fuzzy membership function was adopted to incorporate the impreciseness of data through sustainability assessment by which DMs are able to express their opinion based on subjective preferences instead of using risky deterministic numbers.
➢ DM. However, the importance weights of criteriaThe model led us to identify the weaker sub-criteria that are weakening the sustainability performance in manufacturing systems. In the particular case, eight obstacles were identified out of a total of fifteen sub-criteria. Enhancing the performance of these obstacles will affect the overall sustainability level and improves it.

The summarized fuzzy sustainable manufacturing company index’s calculations includes: select the relevant sub-criteria based on the objectives of organization and determine fuzzy weights, assign linguistic performance based on expert’s judgement, obtain the overall FSMCI based on selected criteria/sub-criteria, match the FSMCI with sustainability status terms using Euclidean distance method to determine the current status of sustainability, calculate fuzzy performance importance index for every single sub-criterion to recognize weak points to improve the sustainability index, and finally modify the process to reach the maximum sustainability.

There are also some limitations of this study that can be mentioned as follows: firstly, the linguistic approach which has been adopted for specifying the weights of criteria operates based on the subjective preference of experts which may cause inaccuracies in the final results. This is suggested to use one of weighting approaches such as AHP or BWM for obtaining the optimal weights. Another limitation to be mentioned is that this study proposes a generic framework for obtaining the status of sustainability. Given that, some criteria might be ignored in some cases. The authors suggest that practitioners first evaluate the criteria for the particular system and then apply the proposed calculations.

## Figures and Tables

**Table 1:** List of vital performance indicators regarding the sustainability of the manufacturing sector.

Category/Indicator	References							
	[56]	[57]	[8]	[16]	[11]	[47]	[22]	[58]
**Social**								
- Employee turnover ratio	√	√		√	√			
- Employee’s training	√	√	√	√	√	√	√	√
- Work intensity	√			√	√			
- Customer’s satisfaction	√		√	√	√			√
- Community involvement	√	√	√	√	√		√	√
- Social cohesion		√		√	√	√		√
- Philanthropy					√		√	
- Health and safety					√	√	√	√
**Economic**								
- Cost	√	√	√	√			√	√
- Quality	√	√		√				
- Flexibility		√		√				√
- Innovation							√	
- Responsiveness		√		√		√		
- Efficiency			√				√	√
- Risk & crisis management							√	
- Employee compensation					√		√	
- Marketing practices							√	
**Environmental**								
- Material Intensity			√	√		√	√	√
- Reused material ratio			√	√			√	√
- Recyclable material ratio			√	√				√
- Hazardous material ratio		√	√	√		√		√
- Waste material ratio		√		√			√	√
- Energy usage		√	√	√		√		√
- Renewable energy ratio			√	√		√		√
- Water consumption	√	√	√	√		√	√	√
- Land usage	√	√		√				√
- Waste water ratio		√	√	√		√	√	√
- Harmful gas missions	√	√	√	√			√	√
- Environmental management						√	√	
**Technological advancement**					√			
- Research and development			√					√
- High tech products			√					
**Performance management**								
- Conformance			√					
- Program and policy			√		√			

**Table 2:** The linguistic terms and corresponding trapezoidal fuzzy numbers.

Performance Ratings		Weight of Criteria	
Linguistic Phrases	Matching Fuzzy Set	Linguistic Phrases	Matching Fuzzy Set
Very weak (VW)	[0,0.25,0.5,1.5]	Not important (NI)	[0,0.025,0.05,0.15]
Weak (W)	[1,1.5,2.5,3]	Weak importance (WI)	[0.1,0.15,0.25,0.3]
Fairly weak (FW)	[2,2.75,4.25,5]	Fairly low importance (FLI)	[0.2,0.275,0.425,0.5]
Intermediate (I)	[3,4,6,7]	Medium importance (MI)	[0.3,0.4,0.6,0.7]
Fairly good (FG)	[5,5.75,7.25,8]	Fairly high importance (FHI)	[0.5,0.575,0.725,0.8]
Good (G)	[7,7.5,8.5,9]	High importance (HI)	[0.7,0.75,0.85,0.9]
Best (B)	[8.5,9,9.75,10]	Utmost importance (UI)	[0.85,0.9,0.975,1]

**Table 3:** List of indicators for the assessment of sustainability in a specific manufacturing company.

Category	Indicator	Relevant?	
Social	Employee turnover ratio	Yes	No
	Employee’s training	Yes	No
	Work intensity	Yes	No
	Customer’s satisfaction	Yes	No
	Community involvement	Yes	No
	Social cohesion	Yes	No
	Philanthropy	Yes	No
	Health and safety	Yes	No
Economic	Cost	Yes	No
	Quality	Yes	No
	Flexibility	Yes	No
	Innovation	Yes	No
	Responsiveness	Yes	No
	Efficiency	Yes	No
	Risk & crisis management	Yes	No
	Employee compensation	Yes	No
	Marketing practices	Yes	No
Environmental	Material Intensity	Yes	No
	Reused material ratio	Yes	No
	Recyclable material ratio	Yes	No
	Hazardous material ratio	Yes	No
	Waste material ratio	Yes	No
	Energy usage	Yes	No
	Renewable energy ratio	Yes	No
	Water consumption	Yes	No
	Land usage	Yes	No
	Waste water ratio	Yes	No
	Harmful gas missions	Yes	No
	Environmental management	Yes	No
Technological advancement	Research and development	Yes	No
	High tech products	Yes	No
Performance management	Conformance	Yes	No
	Program and policy	Yes	No

**Table 4:** The selected sub-criteria and corresponding weights in linguistic form.

Category	Importance	Indicator	Importance
Social	Fairly High (FH)	Employee’s training	Fairly high importance (FHI)
		Work intensity	Utmost importance (UI)
		Customer’s satisfaction	Fairly high importance (FHI)
		Health and safety	Utmost importance (UI)
Economic	Fairly High (FH)	Cost	Utmost importance (UI)
		Quality	Utmost importance (UI)
		Innovation	Fairly high importance (FHI)
		Efficiency	Fairly high importance (FHI)
Environmental	Very High (VH)	Material Intensity	Fairly high importance (FHI)
		Reused material ratio	Medium importance (MI)
		Hazardous material ratio	Fairly high importance (FHI)
		Waste material ratio	Medium importance (MI)
		Energy usage	Utmost importance (UI)
		Waste water ratio	Fairly high importance (FHI)
		Harmful gas missions	Utmost importance (UI)

**Table 5:** The judgements of four DMs concerning the performance of selected sub-criteria.

Category	Indicator	Performance			
		DM1	DM2	DM3	DM4
Social	Employee’s training	Intermediate (I)	Fairly weak (FW)	Good (G)	Fairly weak (FW)
	Work intensity	Intermediate (I)	Weak (W)	Fairly weak (FW)	Fairly weak (FW)
	Customer’s satisfaction	Very Well (VW)	Very Well (VW)	Good (G)	Very Well (VW)
	Health and safety	Intermediate (I)	Fairly weak (FW)	Good (G)	Intermediate (I)
Economic	Cost	Very Well (VW)	Fairly weak (FW)	Best (B)	Fairly weak (FW)
	Quality	Very Well (VW)	Very Well (VW)	Good (G)	Best (B)
	Innovation	Very Well (VW)	Very Well (VW)	Best (B)	Best (B)
	Efficiency	Intermediate (I)	Intermediate (I)	Good (G)	Intermediate (I)
Environmental	Material intensity	Weak (W)	Weak (W)	Fairly weak (FW)	Weak (W)
	Reused material ratio	Weak (W)	Intermediate (I)	Intermediate (I)	Weak (W)
	Hazardous material ratio	Weak (W)	Fairly weak (FW)	Fairly weak (FW)	Fairly weak (FW)
	Waste material ratio	Very Well (VW)	Very Well (VW)	Very Well (VW)	Very Well (VW)
	Energy usage	Weak (W)	Fairly weak (FW)	Good (G)	Good (G)
	Waste water ratio	Intermediate (I)	Fairly weak (FW)	Intermediate (I)	Fairly weak (FW)
	Harmful gas missions	Very Well (VW)	Best (B)	Best (B)	Best (B)

**Table 6:** The fuzzy judgements and the mean of performance.

Indicator	Performance				
	DM1	DM2	DM3	DM4	Mean
Employee’s training	[3,4,6,7]	[2,2.75,4.25,5]	[5,5.75,7.25,8]	[2,2.75,4.25,5]	[3,3.81,5.43,6.25]
Work intensity	[3,4,6,7]	[1,1.5,2.5,3]	[2,2.75,4.25,5]	[2,2.75,4.25,5]	[2,2.75,4.25,5]
Customer’s satisfaction	[7,7.5,8.5,9]	[7,7.5,8.5,9]	[5,5.75,7.25,8]	[7,7.5,8.5,9]	[6.5,7.06,8.18,8.75]
Health and safety	[3,4,6,7]	[2,2.75,4.25,5]	[5,5.75,7.25,8]	[3,4,6,7]	[3.25,4.12,4.56,6.75]
Cost	[7,7.5,8.5,9]	[2,2.75,4.25,5]	[8.5,9,9.75,10]	[2,2.75,4.25,5]	[4.87,5.5,6.68,7.25]
Quality	[7,7.5,8.5,9]	[7,7.5,8.5,9]	[5,5.75,7.25,8]	[8.5,9,9.75,10]	[6.87,7.43,8.5,9]
Innovation	[7,7.5,8.5,9]	[7,7.5,8.5,9]	[8.5,9,9.75,10]	[8.5,9,9.75,10]	[7.75,8.25,9.12,9.5]
Efficiency	[3,4,6,7]	[3,4,6,7]	[5,5.75,7.25,8]	[3,4,6,7]	[3.5,4.43,6.31,7.25]
Material intensity	[1,1.5,2.5,3]	[1,1.5,2.5,3]	[2,2.75,4.25,5]	[1,1.5,2.5,3]	[1.25,1.81,2.93,3.5]
Reused material ratio	[1,1.5,2.5,3]	[3,4,6,7]	[3,4,6,7]	[1,1.5,2.5,3]	[2,2.75,4.25,5]
Hazardous material ratio	[1,1.5,2.5,3]	[2,2.75,4.25,5]	[2,2.75,4.25,5]	[2,2.75,4.25,5]	[1.75,2.43,3.81,4.5]
Waste material ratio	[7,7.5,8.5,9]	[7,7.5,8.5,9]	[7,7.5,8.5,9]	[7,7.5,8.5,9]	[7,7.5,8.5,9]
Energy usage	[1,1.5,2.5,3]	[2,2.75,4.25,5]	[5,5.75,7.25,8]	[5,5.75,7.25,8]	[3.25,3.93,5.31,6]
Waste water ratio	[3,4,6,7]	[2,2.75,4.25,5]	[3,4,6,7]	[2,2.75,4.25,5]	[2.5,3.37,5.12,6]
Harmful gas missions	[7,7.5,8.5,9]	[8.5,9,9.75,10]	[8.5,9,9.75,10]	[8.5,9,9.75,10]	[8.12,8.62,9.43,9.75]

**Table 7:** The detailed data about importance weights and performance rates for this particular case.

Criteria	Sub-Criteria	Importance (Criteria)	Importance (Sub-Criteria)	Performance (Mean)
Social	Employee’s training	[0.5,0.575,0.725,0.8]	[0.5,0.575,0.725,0.8]	[3,3.81,5.43,6.25]
	Work intensity		[0.85,0.9,0.975,1]	[2,2.75,4.25,5]
	Customer’s satisfaction		[0.5,0.575,0.725,0.8]	[6.5,7.06,8.18,8.75]
	Health and safety		[0.85,0.9,0.975,1]	[3.25,4.12,4.56,6.75]
Economic	Cost	[0.5,0.575,0.725,0.8]	[0.85,0.9,0.975,1]	[4.87,5.5,6.68,7.25]
	Quality		[0.85,0.9,0.975,1]	[6.87,7.43,8.5,9]
	Innovation		[0.5,0.575,0.725,0.8]	[7.75,8.25,9.12,9.5]
	Efficiency		[0.5,0.575,0.725,0.8]	[3.5,4.43,6.31,7.25]
Environmental	Material Intensity	[0.85,0.9,0.975,1]	[0.5,0.575,0.725,0.8]	[1.25,1.81,2.93,3.5]
	Reused material ratio		[0.3,0.4,0.6,0.7]	[2,2.75,4.25,5]
	Hazardous material ratio		[0.5,0.575,0.725,0.8]	[1.75,2.43,3.81,4.5]
	Waste material ratio		[0.3,0.4,0.6,0.7]	[7,7.5,8.5,9]
	Energy usage		[0.85,0.9,0.975,1]	[3.25,3.93,5.31,6]
	Waste water ratio		[0.5,0.575,0.725,0.8]	[2.5,3.37,5.12,6]
	Harmful gas missions		[0.85,0.9,0.975,1]	[8.12,8.62,9.43,9.75]

**Table 8:** The obtained performance rates for the criteria level.

Criteria	Importance (Criteria)	Performance (Criteria)
Social	[0.5,0.575,0.725,0.8]	[3.41,4.21,5.42,6.59]
Economic	[0.5,0.575,0.725,0.8]	[5.77,6.28,7.64,8.23]
Environmental	[0.85,0.9,0.975,1]	[3.97,4.57,5.75,6.33]

**Table 9:** Sustainability status terms and corresponding fuzzy sets.

Sustainability Status Terms (SST_*i*_)	Corresponding TrFS
Extremely Sustainable (ES)	[7,7.75,9.25,10]
Very Sustainable (VS)	[5,6.25,7.75,8.5]
Normally Sustainable (NS)	[3.5,4.25,5.75,6.5]
Fairly Sustainable (FS)	[1.5,2.25,3.75,4.5]
Poorly Sustainable (PS)	[0,0.75,2.25,3]

**Table 10:** The obtained distances and determination of the current status.

Sustainability Status Terms (SST*_i_*)	Corresponding TrFS	$\mathrm{Distance}\;\mathrm{Function}\;D\left(FSMCI,\;SLL_{i}\right)$	$\mathrm{Distance}\;\mathrm{Value}\;\left(D_{i}\right)$
Extremely Sustainable (ES)	[7,7.75,9.25,10]	D(FSMCI, ES)	5.78
Very Sustainable (VS)	[5,6.25,7.75,8.5]	D(FSMCI, VS)	2.61
Normally Sustainable (NS)	[3.5,4.25,5.75,6.5]	D(FSMCI, NS)	1.25
Fairly Sustainable (FS)	[1.5,2.25,3.75,4.5]	D(FSMCI, FS)	5.22
Poorly Sustainable (PS)	[0,0.75,2.25,3]	D(FSMCI, PS)	8.22

**Table 11:** The fuzzy performance importance indexes and ranking scores.

Sub-Criteria	Performance	$\begin{array}{l} W_{ij}^{*}\\ =\left[1,1,1,1\right]-W_{ij} \end{array}$	FPII	Ranked FPII
Employee’s training	[3,3.81,5.43,6.25]	[0.2,0.275,0.425,0.5]	[0.6,1.04,2.30,3.12]	1.85
Work intensity	[2,2.75,4.25,5]	[0,0.025,0.1,0.15]	[0,0.06,0.42,0.75]	0.26
Customer’s satisfaction	[6.5,7.06,8.18,8.75]	[0.2,0.275,0.425,0.5]	[1.3,1.94,3.47,4.37]	2.72
Health and safety	[3.25,4.12,4.56,6.75]	[0,0.025,0.1,0.15]	[0,0.10,0.45,1.01]	0.31
Cost	[4.87,5.5,6.68,7.25]	[0,0.025,0.1,0.15]	[0,0.13,0.66,1.08]	0.41
Quality	[6.87,7.43,8.5,9]	[0,0.025,0.1,0.15]	[0,0.18,0.85,1.35]	0.54
Innovation	[7.75,8.25,9.12,9.5]	[0.2,0.275,0.425,0.5]	[1.55,2.26,3.87,4.75]	3.07
Efficiency	[3.5,4.43,6.31,7.25]	[0.2,0.275,0.425,0.5]	[0.7,1.21,2.68,3.62]	1.98
Material intensity	[1.25,1.81,2.93,3.5]	[0.2,0.275,0.425,0.5]	[0.25,0.49,1.24,1.75]	0.88
Reused material ratio	[2,2.75,4.25,5]	[0.3,0.4,0.6,0.7]	[0.6,2,2.7,3.5]	2.3
Hazardous material ratio	[1.75,2.43,3.81,4.5]	[0.2,0.275,0.425,0.5]	[0.35,0.66,1.61,2.25]	1.16
Waste material ratio	[7,7.5,8.5,9]	[0.3,0.4,0.6,0.7]	[2.1,3,5.1,6.3]	4.07
Energy usage	[3.25,3.93,5.31,6]	[0,0.025,0.1,0.15]	[0,0.09,0.53,0.9]	0.33
Waste water ratio	[2.5,3.37,5.12,6]	[0.2,0.275,0.425,0.5]	[0.5,0.92,2.17,3]	1.57
Harmful gas missions	[8.12,8.62,9.43,9.75]	[0,0.025,0.1,0.15]	[0,0.21,0.94,1.46]	0.60

**Table 12:** The weak points and corresponding criteria.

Criteria	Weaker Sub-Criteria	Ranked FPII
Social	Work intensity	0.26
	Health and safety	0.31
Economic	Cost	0.41
	Quality	0.54
Environmental	Material intensity	0.88
	Hazardous material ratio	1.16
	Energy usage	0.33
	Harmful gas missions	0.60
